# Analysis of Technological and Consumption Quality of Offal and Offal Products Obtained from Pulawska and Polish Landrace Pigs

**DOI:** 10.3390/ani10060964

**Published:** 2020-06-01

**Authors:** Marek Babicz, Kinga Kropiwiec-Domańska, Ewa Skrzypczak, Magdalena Szyndler-Nędza, Karolina Szulc

**Affiliations:** 1 Institute of Animal Breeding and Biodiversity Conservation, Faculty of Biology, Animal Sciences and Bioeconomy, University of Life Sciences in Lublin, Akademicka 13, 20-950 Lublin, Poland; marek.babicz@up.lublin.pl; 2 Department of Animal Breeding and Product Quality Assessment, Poznań University of Life Sciences, Złotniki, ul Słoneczna 1, 62-002 Suchy Las, Poland; ewa.skrzypczak@up.poznan.pl (E.S.); karolina.szulc@up.poznan.pl (K.S.); 3 Department of Pig Breeding, National Research Institute of Animal Production, Krakowska 1, Balice n., 32-083 Kraków, Poland; magdalena.szyndler@izoo.krakow.pl

**Keywords:** pigs, Pulawska breed, Polish Landrace breed, offal, offal products, quality

## Abstract

**Simple Summary:**

In many countries, offal is an important culinary and technological raw material used for the production of offal dishes and products. Given the growing popularity of such products, as well as insufficient knowledge about the technological and nutritional value of offal, it is important to determine the properties of some pork offal and offal products. The study material consisted of 100 fattening pigs: 50 Pulawska pigs and 50 Polish Landrace pigs. The offal components (tongue, heart, lungs, liver, kidneys) were analysed for physical traits, basic chemical composition and energy value. Offal products (pate, liver sausage, brawn) were made from the offal and their physical, chemical and organoleptic parameters were evaluated. The results, obtained in our study, indicate a high technological and consumption quality of offal from Pulawska and Polish Landrace pigs. The organoleptic quality of the evaluated offal products was higher for Pulawska than Polish Landrace pigs, in particular with regard to aroma and flavour. We suggest that our study could influence consumer awareness of the consumption of pork offal and offal preparations while increasing the use for production of the Pulawska breed, and thus, other native breed characteristic of many other countries of the world.

**Abstract:**

The aim of the study was to determine technological and consumption quality of some offal components obtained from Pulawska and Polish Landrace fattening pigs, and to analyse the eating quality of the offal products. The study material consisted of 100 fattening pigs: Pulawska (PUL) and 50 Polish Landrace (PL) pigs. The offal components were analysed for physical traits, chemical composition and energy value. Offal products were made from the offal and their physical, chemical and organoleptic parameters were evaluated. Our study showed that breed had a significant effect (*p* ≤ 0.05) on pH_45_ of the tongue, heart (PUL > PL), lungs and kidneys (PUL < PL), and on the fat content of the tongue, heart (PUL > PL), liver and kidneys (PUL < PL). A highly significant effect of breed (*p* ≤ 0.01) was observed for protein content of the lungs, liver (PUL < PL) and kidneys (PUL > PL), for collagen content of the kidneys (PUL < PL) and liver (PUL < PL), and for energy value (*p* ≤ 0.01) of the heart (PUL > PL) and liver (PUL < PL). Moreover, our results indicate that the organoleptic quality of the evaluated offal products was higher for Pulawska than Polish Landrace pigs, in particular with regard to consistency (*p* < 0.05) and flavour (*p* < 0.01) of the liver sausage.

## 1. Introduction

Offal is an important component of the meat industry. The main factors affecting the volume of offal consumption around the world include consumer preferences, income, cultural zone, and religion. Offal is the basic ingredient of traditional dishes consumed in many countries of the world [1]. In Spain, Italy, Egypt, Republic of South Africa and Asian countries, all slaughterhouse meat by-products (including spleen, pancreas and uterus) are commonly used in human nutrition. Some countries differ in the consumption of these products according to the animal species they come from, for example, poultry offal is most often eaten in Japan, and goat offal in India, Indonesia, Bangladesh and Pakistan [2]. Pork offal is most frequently used for production of offal products, i.e. pate, liver sausage, black pudding, brawn, and canned offal [3,4,5]. The production of food products, containing offal, increases the potential for their use, and thus, enhances the commercial value of the offal. This is directly reflected in an expanded range of pork products for the consumer, as well as influencing the economic performance of meat processing plants [6]. 

It is accepted that the quality of offal, as in the case of pork, depends on genetic factors, of which the most important is breed [7,8,9,10]. In this context, it is important to highlight an upward global trend of using native breeds for the production of meat and cured meats. In Poland, this applies to the Pulawska, Zlotnicka White and Zlotnicka Spotted breeds. 

The Pulawska breed is native to the Lublin region and breeding tradition dates back to 1926. The breed was created by crossing the primitive long-eared, small prick-eared and Berkshire breeds. Selective breeding in the 1930s resulted in a population of lard-type pigs, known as Golebska. In 1951, the name was replaced with Pulawska. In the 1980s and 1990s, Pulawska breeding suffered a setback due to lower muscling parameters, compared to those of imported meat-type breeds (Pietrain, Hampshire, Duroc). In 1996 the breed was included in the genetic resources conservation programme [11]. Today, the population shows slaughter traits, characteristic of the fatty-meat type, and is used to produce high quality meat and superior cured meats. The native Polish Landrace is a meat breed used for large-scale production of slaughter raw materials with desired technological quality. 

The Polish Landrace breed represents the Landrace type of pigs popular around the world. The breed originated from domestic floppy-eared pigs and German Improved pigs of the Westphalian type. In 1961, the population of landrace pigs raised in Poland was recognized as the Polish Landrace breed. In the 1970s, lop-eared pigs were imported into Poland from other European countries and kept as separate lines: pbz 21—Norwegian line, pbz 22—Dutch line, pbz 23—German line, pbz 24—Welsh line. Since 1992, Polish Landrace pigs and animals of the above lines were kept as a single breeding population. The Pulawska breed differs from the Landrace breed in rearing conditions. Pulawska pigs are well adapted to local environmental conditions. Moreover, from the earliest days of breeding, they have been raised in family farms in high welfare conditions and fed based on farm-produced feeds The Polish Landrace breed is raised in the intensive production system and fed based on cereals and commercial mixtures. 

The present study compared these breeds with regard to different breeding traditions and performance levels. Pulawska pigs are the fatty-meat type, whereas Polish Landrace pigs are of the meat type. Therefore, the slaughter raw material from these breeds may differ in technological and eating quality [12].

As reported by Prasow et al. [13], compared to Polish Landrace meat, the meat of Pulawska pigs is characterized by darker colour, better pH and water holding capacity (WHC) values, lower drip loss and cooking loss, lower protein and cholesterol content, and higher intramuscular fat content. Today, there is little scientific information available concerning the characteristics of offal obtained from these breeds [14,15].

The aim of the study was to determine technological and consumption quality of some offal components obtained from Pulawska and Polish Landrace fattening pigs, and to analyse the eating quality of the offal products. 

It was our hypothesis that breed of fatteners influences the quality of pork offal, which in turn determines the quality of offal products.

## 2. Materials and Methods 

### 2.1. Experimental Procedures

#### Animals

This study did not require Ethics Committee approval. The experiment was conducted with 100 fattening pigs, including 50 Pulawska pigs (PUL group) and 50 Polish Landrace pigs (PL group), with an equal number of boars and gilts. Pigs were kept in littered floor pens in compliance with animal welfare requirements [16]. At the start of the study, weaners were aged 11–12 weeks and their body weight was 25 kg (±2kg). Single-phase dry feeding was used. Pigs were fed a diet containing 15% protein, 2.2% fat, 5.5% fibre and 12.7 MJ Metabolic Energy, which was offered ad libitum via standard tube feeders.

Fattening results are presented in Table 1.

Pigs were slaughtered at a Meat Processing Plant after reaching 112 kg (±3 kg) body weight. Fatteners were transported to the meat plant up to a distance of 180 km, in accordance with the standards laid down in Council Regulation (EC) No 1/2005 of 22 December 2004 on the protection of animals during transport and related operations [17]. Animals were slaughtered around 3 h after the transport, in accordance with company standards, using automatic electrical stunning (250V, 5A, 2.4s) and exsanguination in a lying position.

The main indicators of slaughter value are shown in Table 1. 

### 2.2. Preparation of Samples for Laboratory Analysis

Tongue, heart, lungs, liver and kidneys were sampled from each pig. The choice of offal was determined by consumer acceptance. The internal organs were rinsed under running water to remove blood clots and possible bone particles. Next, visible connective and adipose tissue was carefully separated from offal. Tongues were obtained by cutting the hyoid bone and removing the larynx. Hearts were trimmed to clean the atria and ventricles from blood clots and to remove residual aorta and pulmonary vein. The lung was separated from the trachea. The liver was separated from the gallbladder, hepatic lymph nodes, hepatic artery and portal vein. Kidneys were released from the adipose capsule, after which residual urinary apparatus (ureter) and vascular system (renal vein and artery) were cut from the renal hilus. Cleaned offal was weighed, placed in portable refrigerators (temperature +4 °C) and transported to a laboratory, where it was kept under cold storage conditions at +4 °C.

#### Determination of Physical Properties and Chemical Composition of the Offal

The analysis of physical traits included measuring the offal for the concentration of hydrogen ions 45 min and 24 h postmortem with a pH-Star CPU device (Matthäus), which is used in the meat plant in which the animals were slaughtered. Moreover, the percentage of free water (WHC—water holding capacity) was determined by the method of Grau and Hamm [18] modified by Pohja and Niinivaara [19].

The offal samples were homogenized 48 h postmortem using a Büchi Mixer B-400 (Flawil, Switzerland) and analysed for the percentage of water, total protein, collagen, and total fat. This was done with a FoodScanTM meat analyser (Foos) using near-infrared spectrometry (NIR) according to standard PN-A-82109 [20].

The energy value (kJ per 100 g^−1^ of fresh tissue) of each offal was calculated from the total protein and fat percentage. The calculations were based on Atwater equivalents, where: 1 g protein = 4.0 kcal = 16.76 kJ; 1 g fat = 9.0 kcal = 37.66 kJ. 

### 2.3. Production and Evaluation of Offal Products

The obtained offal was used to make offal products (pate, liver sausage, brawn) according to the formulas and procedures used in the meat processing plant. The basic ingredients of the offal products were as follows: pate (pork meat gread II A and II B, pork rinds, liver, dried vegetables, natural seasonings, salt, water); liver sausage (lungs, kidneys, liver, pork rinds, semolina, dried vegetables, natural seasonings, salt, water); brawn (tongues, hearts, liver, pork head meat, dried vegetables, natural seasonings, salt, water). The products were evaluated 48 h after manufacture. The same methods as for raw offal were used to determine physical traits (pH, WHC) and chemical composition (water, protein, collagen, fat), and the energy value was calculated. 

Consumer sensory evaluation of the offal products was carried out using score cards for external appearance, colour, aroma, consistency, flavour, juiciness and overall score. Panelists were asked to score their overall liking of a trait (qualitative assessment). Offal products were assigned to one of the following quality characteristics: 5—very good, 4—good, 3—adequate, 2—poor, 1—very poor. The consumer panel consisted of 40 people (20 women and 20 men) aged between 19 and 26 years. All panelists were trained on how to perform the evaluation and instructed how to understand the assessed traits. 

### 2.4. Statistical Analysis

The analyses were performed using the SAS 9.4 software (SAS Institute Inc. Cary, NC, USA) for analysis of data. The normality was assessed using the Kolmogorov–Smirnov test, and the Levene’s homogeneity of variance test was applied to examine the equality of variances. The general linear model (GLM) procedure for analyses of variance included breed. Tukey’s test was applied for the multiple comparisons among means, considering *p* ≤ 0.05 as significant and *p* ≤ 0.01 as highly significant.

The linear model formula was as follows,

y_ij_ = µ + b_i_ + e_ij_

where: y_ij_—phenotypic value of a trait observation of ij-th individual; µ—population mean; b_i_ —fixed effect of pig breed (I = 1, 2); e_ij_—random error.

## 3. Results and Discussion

The main parameter determined for the analysed offal was its weight and live weight percentage (Table 2). The weights of different offals (Table 2) were similar to the data presented in the Encyclopedia of Meat Sciences [21] and those reported by Kaswan et al. (2016) [22]. Numerical data for percentage of different offals in live weight of the pigs are consistent with those of other authors [23]. The statistical analysis of the effect of breed on weight and live weight percentage demonstrated significant (*p* ≤ 0.01) differences only for liver. The weight of liver, obtained from Pulawska pigs, was 0.14 kg higher than for Polish Landrace fatteners. The pH and WHC values provide information concerning the rate of postmortem change in offal [24]. Statistically significant differences in pH_45_ between the Pulawska and Polish Landrace breeds were observed for the tongue (PUL > PL, *p* ≤ 0.01), heart, kidneys, (PUL < PL, *p* ≤ 0.01) and lungs (PUL < PL, *p* ≤ 0.05) (Table 3). For pH_24_, a significant (*p* ≤ 0.05) effect of breed was observed only for kidneys (Table 3). It can be assumed that the different pH values in different internal organs are due to their distinct histological structure and physiological role. The differences in pH may also result from biochemical processes associated with the maintenance of acid-base balance. The value noted for the PL group was higher by 0.2 compared to the PUL group. In both breed groups, significantly (*p* ≤ 0.01) lowest pH_24_ values were observed for the tongue, and highest for the lungs, which is consistent with the findings of Tomović et al. [25]. We found a downward trend for the tongue and heart, and an upward trend for the lungs, liver and kidneys, when comparing pH_24_ and pH_45_ values of the offal from Polish Landrace and Pulawska breeds. It is necessary to stress that the logarithm of hydrogen ion concentration (pH) determines microbiological stability and technological properties of slaughter material [26]. The postmortem changes, expressed as pH measured 45 min and 24 h after slaughter, in the tongues and in the hearts of the PL and PUL groups show similarity to those occurring in normal meat (RFN) [27,28]. This distribution of values may be attributed to the similar histological structure of the heart (striated cardiac muscle tissue), tongue (striated skeletal muscle tissue) and meat (striated skeletal muscle tissue).

Water holding capacity determines the organoleptic properties of slaughter raw materials and slaughter products by influencing texture, tenderness, and good binding [29]. Our study showed no effect of pig breed on WHC of the offal (Table 3).

In terms of chemical composition, the main component of offal, is water, followed by protein and fat (Table 3) According to Pearce et al. [30], it determines the quality and storage life of slaughter material. Of the analysed offal, the lowest water content (Table 3) was characteristic of liver in both breed groups and this information agrees with the data presented by Seong et al. [23]. Around 5% higher water proportion in pork liver was reported by Kim et al. [31]. In our study, water content of lungs was over 4 percentage points higher (*p* ≤ 0.05) in Pulawska pigs compared to Polish Landrace pigs. The water content of the tongue, heart and kidneys in both breeds from our study is lower than the values reported for pork offal by Seong et al. [23], but also lower than for veal offal [32]. These values were close to the water content of pork meat, i.e., around 73% [33,34,35]. 

The protein content correlates with the sensory and technological properties of pork products [36]. Analysis of protein content (Table 3) in the offal of Polish Landrace and Pulawska pigs showed that it was highest in the liver of both study groups, which agrees with the results of other authors [23,25,37]. Comparative analysis of the protein content of offal with the data reported by the US Department of Agriculture’s Food Data Central [38] showed the highest consistency for the tongue and heart of both groups, and for the liver of the PUL group. 

When comparing our values for offal from the conservation breed of Pulawska pigs with the offal data for the autochthonous Swallow-Belly Mangalitsa breed from Hungary [25], we found similar protein content for the tongue, heart and liver. Breed had a statistically significant effect on the protein content of pork offal. Compared to the Polish Landrace breed, Pulawska fatteners contained less protein in the lungs and liver (*p* ≤ 0.01) and more protein in the kidneys (*p* ≤ 0.01). 

One of the proteins with a considerable impact on the quality of pork raw materials is collagen. Although it is an incomplete protein source [39], it has a significant effect on tenderness, a trait highly desired by the consumer [40]. Protein digestibility decreases with increasing collagen content of offal, which makes the offal less tender and lowers its nutritional value [41]. The collagen content of the analysed offal (Table 3) ranged from 1.28% (liver in the PUL group) to 3.30% (lungs in the PL group). The observed differences in the offal collagen content between the two breeds were statistically significant (*p* ≤ 0.01). The effect of breed (*p* ≤ 0.01) on the pork offal collagen content was noted for kidneys (PUL < PL) and liver (PUL < PL).

Another chemical component influencing the technological, nutritional and eating quality of slaughter raw materials is fat [42]. Our study revealed that fat content was highest in the tongues from both breed groups. The least fat was found in lungs for the Polish Landrace breed, and in liver for the Pulawska breed. Significant differences (*p* ≤ 0.05) between the PUL and PL groups were observed for all the analysed offals except the lungs (Table 3). The Pulawska breed exhibited a significantly higher fat content in the tongue and heart, and a significantly lower fat content in the liver and kidneys.

Comparison of data in Table 3 with those from the Food Data Central [38] showed that the heart, lungs and liver from group PL as well as kidneys from group PUL were characterized by the closest fat content to the reference values reported there. Similar relationships were observed when comparing our data with those of Kunachowicz et al. [37] and Seong et al. [23]. The proportion of fat in the lungs of the PL and PUL groups, in the liver of the PL group, and in the kidneys of the PUL group is comparable to that in the tongue and heart of both groups lower than, the values reported by Tomović et al. [25].

The amount of fat and protein is directly related to the energy value of offal. In our study, the effect of breed (*p* ≤ 0.01) on energy value of the offal was observed for the heart (PUL > PL) and liver (PUL < PL). Moreover, the highest caloric value of the offal components was characteristic of the tongue in both breed groups (Table 3). The lowest caloric value was noted for the kidneys of Polish Landrace fatteners. Likewise, Florek et al. [32] found that the tongue had the highest energy value out of all the veal offal under analysis. It should be stressed, however, that pork offal has a higher energy value compared to veal or beef offal, as reported by Honikel [43]. The energy value of the liver, heart and lungs from the pigs of both breeds was lower than that presented by Seong et al. [23].

The quality of cured meats as the end product depends primarily on the technological and eating quality of the ingredients used for their production [44]. 

Table 4 presents the physical and chemical parameters of the analysed offal products. The differences observed between the breeds in the content of physichochemical components in the analysed offal products were not significant, which is indicative of the good quality of the raw material from which the offal products were made.

With respect to chemical composition, the liver sausage from the PUL group contained more protein and less fat compared to the PL group, which could be beneficial for those trying to reduce the intake of fat. The pate and brawn from the group of Pulawska pigs, compared to Polish Landrace fatteners, had a lower protein and higher fat content, which is beneficial as far as sensory traits are concerned.

The offal products were subjected to consumer evaluation and the results are shown in Table 5. External appearance, colour, consistency, flavour and juiciness were assessed, and the overall quality evaluation was presented. Among the manufactured offal products, the highest scores in both study groups were given to pate, followed by brawn and liver sausage.

Analysis of the consumer evaluation of pate showed that the pate made from Pulawska pig offal received higher scores for all the tested parameters except juiciness. 

The scores awarded for different brawn traits ranged from 4.08 (flavour, PL group) to 4.29 (juiciness, PL group). The brawn made from Pulawska pig offal received a higher total evaluation score compared to the brawn made from Polish Landrace pig offal.

In the case of liver sausage, statistically significant differences between the PL and PUL groups were observed for consistency (*p* < 0.05) and flavour (*p* < 0.01).

Consumers who buy offal products in stores use their senses to assess the external appearance, colour, possibly aroma. Consistency, juiciness and taste of an offal product are the components evaluated during its consumption and determine whether the consumer will purchase it again [45]. 

## 4. Conclusions

The present study showed that pig breed has an effect on physical and chemical indicators of pork offal. Analysis of the physical properties of the offal obtained from Pulawska and Polish Landrace fattening pigs showed no adverse changes within 24 h post-mortem, which confirms the high technological quality of the offal. With respect to consumption quality, breed influenced the protein, collagen and fat content of the offal, and had an effect on the energy value. In general, the offal products, produced in this study, showed favourable consumption quality. All the offal products were positively evaluated by consumers. The products made from the offal of Pulawska fatteners received higher overall scores for quality compared to the products made from the offal of Polish Landrace pigs. 

## Figures and Tables

**Table 1:** Fattening results and slaughter value parameters of experimental pigs (mean ± SD).

Traits	Group	
	PL	PUL
Daily gains during fattening, g	965.52 ± 45.22	810.33 ± 37.34
Duration of fattening, days	90.1 ± 3.81	107,36 ± 4.21
Backfat thickness, mm	9.82 ± 0.97	12.73 ± 1.02
Loin eye height, mm	59.5 ± 2.33	52.26 ± 2.11
Meat content, %	59.65 ± 3.14	56.11 ± 2.76
Dressing percentage, %	0.78 ± 0.03	0.76 ± 0.02

PL—Polish Landrace pigs; PUL—Pulawska pigs.

**Table 2:** Weight and percentage of different offals in live weight of pigs (mean ± SD).

Traits	Group	Offal Type									
		Tongue	Breed *p*-Value	Heart	Breed *p*-Value	Lungs	Breed *p*-Value	Liver	Breed *p*-Value	Kidney	Breed *p*-Value
Weight, kg	PL	0.25 ± 0.03	NS	0.40 ± 0.03	NS	0.67 ± 0.11	NS	1.27 ± 0.21	**	0.17 ± 0.02	NS
	PUL	0.28 ± 0.01		0.39 ± 0.01		0.71 ± 0.08		1.41 ± 0.11		0.19 ± 0.02	
Live weight, %	PL	0.23 ± 0.02	NS	0.36 ± 0.03	NS	0.60 ± 0.10	NS	1.14 ± 0.20	**	0.15 ± 0.01	NS
	PUL	0.25 ± 0.01		0.35 ± 0.01		0.64 ± 0.08		1.25 ± 0.09		0.17 ± 0.02	

**—Significant differences between breeds (*p* ≤ 0.01); NS—not significant.

**Table 3:** Physical and chemical parameters of the evaluated offal depending on pig breed (mean ± SD).

Traits	Group	Offal Type									
		Tongue	Breed *p*-Value	Heart	Breed *p*-Value	Lungs	Breed *p*-Value	Liver	Breed *p*-Value	Kidney	Breed *p*-Value
			
pH_45_	PL	6.05 ± 0.24	**	6.40 ± 0.26	**	6.70 ± 0.19	*	6.00 ± 0.28	NS	6.29 ± 0.15	**
	PUL	6.27 ± 0.18		6.16 ± 0.19		6.51 ± 0.23		6.11 ± 0.25		6.05 ± 0.11	
pH_24_	PL	5.67 ± 0.21	NS	6.13 ± 0.32	NS	6.90 ± 0.23	NS	6.08 ± 0.15	NS	6.58 ± 0.13	*
	PUL	5.75 ± 0.18		5.97 ± 0.20		6.84 ± 0.27		6.25 ± 0.22		6.38 ± 0.16	
WHC, %	PL	14.49 ± 1.77	NS	14.51 ± 1.41	NS	14.43 ± 0.85	NS	20.82 ± 2.95	NS	12.89 ± 1.36	NS
	PUL	14.99 ± 2.15		14.01 ± 2.36		14.34 ± 0.62		19.88 ± 1.53		11.94 ± 1.53	
Water, %	PL	69.19 ± 2.68	NS	74.63 ± 2.34	NS	74.90 ± 6.57	*	64.32 ± 1.68	NS	76.57 ± 3.57	NS
	PUL	67.47 ± 2.03		73.62 ± 2.35		78.96 ± 5.84		65.60 ± 2.51		75.84 ± 3.09	
Protein, %	PL	17.06 ± 1.19	NS	17.08 ± 1.01	NS	21.16 ± 2.38	**	26.31 ± 1.32	**	13.47 ± 2.17	**
	PUL	16.17 ± 0.60		16.65 ± 0.84		19.26 ± 2.23		21.64 ± 1.86		18.87 ± 3.36	
Collagen, %	PL	2.79 ± 0.25	NS	2.22 ± 0.26	NS	3.30 ± 0.41	NS	1.77 ± 0.22	**	1.80 ± 0.19	**
	PUL	2.81 ± 0.31		2.13 ± 0.34		3.16 ± 0.27		1.28 ± 0.27		1.46 ± 0.22	
Fat, %	PL	12.45 ± 1.36	*	4.28 ± 0.54	*	2.97 ± 0.56	NS	3.85 ± 0.43	*	4.93 ± 0.75	*
	PUL	13.71 ± 1.49		5.77 ± 0.48		3.07 ± 0.41		1.76 ± 0.60		3.12 ± 0.72	
Energy value, kj/100g	PL	754.8 ± 54.0	NS	447.4 ± 20.9	**	466.3 ± 53.5	NS	585.9 ± 29.2	**	411.4 ± 30.2	NS
	PUL	787.4 ± 54.8		496.4 ± 21.2		438.5 ± 48.2		429.1 ± 44.2		433.6 ± 54.2	

pH—negative logarithm of hydrogen ion activity; WHC—Water Holding Capacity; PL—Polish Landrace pigs; PUL—Pulawska pigs. **—Significant differences between breeds (*p* ≤ 0.01); * —Significant differences between breeds (*p* ≤ 0.05); NS—not significant.

**Table 4:** Physical indicators and chemical composition of offal products depending on pig breed (mean ± SD).

Traits	Group	Pate	Brawn	Liver Sausage
pH	PL	6.47 ± 0.05	6.72 ± 0.04	6.56 ± 0.03
	PUL	6.51 ± 0.04	6.58 ± 0.04	6.48 ± 0.02
WHC, %	PL	11.66 ± 0.08	17. 24 ± 0.10	8.57 ± 0.05
	PUL	10. 97 ± 0.07	17. 17 ± 0.11	8.33 ± 0.04
Water, %	PL	51.59 ± 0.33	57.67 ± 0.25	66.39 ± 0.37
	PUL	54.62 ± 0.29	58.19 ± 0.22	61.89 ± 0.34
Protein, %	PL	12.83 ± 0.10	14.13 ± 0.09	14.58 ± 0.10
	PUL	9.38 ± 0.07	12.33 ± 0.08	14.96 ± 0.09
Collagen, %	PL	3.80 ± 0.01	4.52 ± 0.02	2.75 ± 0.01
	PUL	3.29 ± 0.02	4.88 ± 0.03	2.62 ± 0.01
Fat, %	PL	24.66 ± 0.16	23.50 ± 0.17	18.28 ± 0.13
	PUL	33.01 ± 0.22	24.01 ± 0.10	14.12 ± 0.11

pH—negative logarithm of hydrogen ion activity; WHC—Water Holding Capacity; PL—Polish Landrace pigs; PUL—Pulawska pigs.

**Table 5:** Consumer evaluation of the offal products (mean ± SD).

Traits	Group	Pate	Breed *p*-Value	Brawn	Breed *p*-Value	Liver Sausage	Breed *p*-Value
External appearance	PL	3.94 ± 0.96	NS	4.19 ± 0.87	NS	3.49 ± 0.70	NS
	PUL	4.01 ± 0.95		4.14 ± 0.92		3.38 ± 0.68	
Colour	PL	3.95 ± 0.88	NS	4.11 ± 0.70	NS	3.37 ± 0.79	NS
	PUL	4.05 ± 0.79		4.13 ± 0.70		3.32 ± 0.84	
Aroma	PL	4.16 ± 0.90	NS	4.21 ± 0.63	NS	3.51 ± 1.02	NS
	PUL	4.20 ± 0.90		4.26 ± 0.64		3.53 ± 1.00	
Consistency	PL	4.00 ± 1.00	NS	4.28 ± 0.63	NS	3.61 ± 0.73	*
	PUL	4.06 ± 0.97		4.29 ± 0.75		4.10 ± 0.76	
Flavour	PL	4.04 ± 0.87	NS	4.08 ± 0.71	NS	3.43 ± 0.68	**
	PUL	4.21 ± 0.64		4.18 ± 0.78		4.29 ± 0.48	
Juiciness	PL	4.18 ± 0.65	NS	4.23 ± 0.70	NS	3.76 ± 0.67	NS
	PUL	4.15 ± 0.56		4.29 ± 0.72		4.04 ± 0.64	
Total evaluation score	PL	4.19 ± 0.64	NS	4.14 ± 0.58	NS	3.73 ± 0.61	NS
	PUL	4.30 ± 0.58		4.24 ± 0.59		4.12 ± 0.56	

**—Significant differences between breeds (*p* ≤0.01); *—Significant differences between breeds (*p* ≤ 0.05); NS—not significant;
